# Clinical Characteristics for the Improvement of Cushing's Syndrome Complicated With Cardiomyopathy After Treatment With a Literature Review

**DOI:** 10.3389/fcvm.2021.777964

**Published:** 2021-12-01

**Authors:** Sisi Miao, Lin Lu, Ling Li, Yining Wang, Zhaolin Lu, Huijuan Zhu, Linjie Wang, Lian Duan, Xiaoping Xing, Yong Yao, Ming Feng, Renzhi Wang

**Affiliations:** ^1^Key Laboratory of Endocrinology of National Health Commission, Department of Endocrinology, Peking Union Medical College Hospital, Chinese Academy of Medical Science and Peking Union Medical College, Beijing, China; ^2^School of Clinical Medicine, Guizhou Medical University, Guiyang, China; ^3^Department of Cardiology, Peking Union Medical College Hospital, Chinese Academy of Medical Science and Peking Union Medical College, Beijing, China; ^4^Department of Radiology, Peking Union Medical College Hospital, Chinese Academy of Medical Science and Peking Union Medical College, Beijing, China; ^5^Department of Neurosurgery, Peking Union Medical College Hospital, Chinese Academy of Medical Science and Peking Union Medical College, Beijing, China

**Keywords:** Cushing's syndrome, cardiomyopathies, hypercortisolism, cardiac structure, cardiac function

## Abstract

**Background:** Endogenous Cushing's syndrome (CS), also called hypercortisolism, leads to a significant increase in mortality due to excessive cortisol production, which is mainly due to cardiovascular disease. CS complicated with cardiomyopathies, which is a rare and severe condition, has rarely been reported in the literature.

**Objective:** To investigate the clinical characteristics of CS complicated with cardiomyopathies, we retrospectively reviewed the clinical manifestations, laboratory results, cardiac imaging results and prognosis to further understand the diagnosis, treatment, and management of these cases.

**Methods:** The clinical data of patients diagnosed with CS complicated with cardiomyopathies obtained from discharge sheets from Peking Union Medical College Hospital from January 1986 to August 2021 were collected. Case reports of CS complicated with cardiomyopathies were retrieved from PubMed. In addition, Cushing's disease (CD) patients without cardiomyopathies were collected as controls to compare the clinical features.

**Results:** A total of 19 cases of CS complicated with cardiomyopathies and cases of CD without cardiomyopathies (*n* = 242) were collected. The causes of CS included pituitary adenoma (*n* = 8, 42.11%), adrenal adenoma (*n* = 7, 36.84%), ectopic adrenocorticotropic hormone (ACTH) tumor (*n* = 2, 10.53%) and unclear causes (*n* = 2, 10.53%) in the CS complicated with cardiomyopathies group. The types of cardiomyopathies were dilated cardiomyopathies (*n* = 15, 78.94%) and hypertrophic cardiomyopathies (*n* = 4, 21.05%). The serum sodium concentration was significantly higher [145.50 (140.50–148.00) mmol/L vs. 141.00 (140.00–143.00) mmol/L], while the serum potassium concentration was significantly lower [2.70 (2.40–3.60) mmol/L] vs. 3.90 (3.50–4.20 mmol/L)] in the CS complicated with cardiomyopathies group compared to the CD patients without cardiomyopathies. There were no significant differences between the CS complicated with cardiomyopathies group and the CD patients without cardiomyopathies in the serum cortisol concentration and 24-h urine free cortisol, but a significant difference in the adrenocorticotropic hormone level [109.00 (91.78–170.30) pg/ml vs. 68.60 (47.85–110.00) pg/ml]. Twelve/16 (75.0%) patients showed significant improvement or even a complete healing of the heart structure and function after remission of hypercortisolemia after treatment with CS.

**Conclusions:** CS complicated with cardiomyopathies is a very rare clinical entity, in which cortisol plays an important role and it can be greatly improved after remission of hypercortisolemia.

## Introduction

Cushing's syndrome (CS) is a rare disease, which caused by a variety of etiologies and is characterized by excessive cortisol production by the adrenal glands due to a pituitary adenoma, ectopic adrenocorticotropin syndrome secondarily or by adrenal tumor, or autonomous hyperplasia. CS can be complicated by several metabolic abnormalities, such as hypertension, impaired glucose tolerance, and hyperlipidemia, and patients with CS have a high risk of opportunistic infection and coagulation problems due to hypercortisolemia. The mortality in CS is increased significantly with a standard mortality rate (SMR) of 3.68 (2.34–5.33) (1). A study of adrenal incidentaloma with a mean follow-up of 7.5 years showed that, compared with patients with non-secretory adrenal incidentaloma, patients with subclinical hypercortisolism had a nearly four-fold increase in all-cause mortality (8.8 vs. 43%), a 10% higher incidence of cardiovascular events (6.7 vs. 16.7%) and a 7.6 times higher incidence of cardiovascular mortality (2.5 vs. 21.6%) (2). In addition, compared with the patients with a cortisol level <1.8 μg/dL after the dexamethasone suppression test, the patients with a cortisol level between 1.85 and 5 μg/dL had a higher mortality hazard ratio of 12.0 (1.6–92.6), and patients with a cortisol level >5 μg/dL had the highest mortality hazard ratio of 22.0 (2.6–188.3). Notably, 50% of deaths were due to cardiovascular disease (3). Because CS is often diagnosed months or years after the onset of symptoms, an increased risk of cardiovascular disease could be present even before the diagnosis (4).

Given the significantly increased risk of death and cardiovascular disease, several studies have assessed the changes in the cardiac structure and function in CS. Patients with CS often have an increased left ventricular mass and relative wall thickness (5), a higher proportion of impaired diastolic function (6), and a lower left ventricular ejection fraction (LVEF) (7). Several studies investigated whether the cardiac structure and function could improve after remission of hypercortisolism. Toja et al. (5) found that cardiac mass abnormalities (such as an increased left ventricular mass and an increased relative wall thickness) were improved but not completely recovered after curative treatment in CS. Moreover, although the LVEF was within the normal range after remission of CS, the value was still slightly lower than that of the control group. Peter Kamenický et al. (7) found that the subclinical cardiac systolic dysfunction was recovered and the LVEF increased by 15% after remission of CS.

Cardiomyopathies are a group of heterogeneous myocardial diseases characterized by cardiomyocyte disorders, which lead to myocardial mechanical and/or electrical dysfunction. Based on the anatomy and physiology, cardiomyopathies consist of the following types (each with a variety of different causes): dilated cardiomyopathies (DCM), hypertrophic cardiomyopathies (HCM), restrictive cardiomyopathies (RCM), arrhythmic right ventricular cardiomyopathies/dysplasia (ARVC/D) and undefined cardiomyopathies. Since CS is a rare disease with an incidence of 2–5/10^6^/year, CS complicated with cardiomyopathy is rarely seen among the known causes. A search of the literature found that there were only a few case reports about CS complicated with cardiomyopathies, in which the major type was DCM (8–19). If CS induces cardiomyopathies, it is speculated that the mortality risk of CS patients will be further aggravated. To investigate the clinical characteristics of CS complicated with cardiomyopathies, this study described the diagnosis, treatment, management and prognosis of patients with CS complicated with cardiomyopathies in Peking Union Medical College Hospital and a literature review is also discussed.

## Subjects and Methods

### Subjects

#### CS Complicated With Cardiomyopathies in Peking Union Medical College Hospital

The data from the CS cases complicated with cardiomyopathies were obtained through a search of the discharge sheet from the hospital information system at Peking Union Medical College Hospital from January 1986 to August 2021. Written informed consent was obtained from the patients or their family members for the publication of the images and data. The Ethics Committee of Peking Union Medical College Hospital approved this retrospective study.

The inclusion criteria were as follows: (1) a diagnosis of CS, including the typical clinical manifestations of CS, increased midnight cortisol or 24-h urine free cortisol (UFC), and no suppression of cortisol after the low-dose dexamethasone suppression test; and (2) evidence of a cardiomyopathy: an echocardiography or a cardiac magnetic resonance imaging (MRI) showing ventricular dilation and decreased myocardial contractility or left ventricular wall thickening (interventricular septum dimension or left ventricular posterior wall dimension ≥15 mm).

The exclusion criteria were as follows: (1) an enlarged left ventricle and decreased myocardial systolic function resulting from other identified causes, such as long-term poorly controlled hypertension, diabetes, valvular heart disease, congenital or ischemic heart disease; (2) left ventricular wall thickening caused by other identified causes, such as hypertension, aortic stenosis, or congenital subaortic septum; (3) infectious disease; (4) cardiomyopathies diagnosed prior to the onset of CS symptoms; (5) familial cardiomyopathies; (6) systemic autoimmune disease; (7) evidence of amyloidosis; (8) thyroid disorders without treatment; and (9) lack of clinical information.

Altogether, 7 patients who met the enumerated inclusion/exclusion criteria were enrolled. We collected information about these cases, including (1) the demographic features such as age and sex; (2) the time of onset of CS; (3) the clinical symptoms of cardiomyopathies such as dyspnea, fatigue, edema; (4) the physical examination data, such as height, weight, body mass index (BMI), and blood pressure; (5) the laboratory examinations, including routine blood tests, blood glucose, serum potassium, serum sodium, blood lipids, and hypersensitive C-reactive protein; (6) the pituitary and adrenal hormone tests, including adrenocorticotrophic hormone (ACTH), serum cortisol, 24-hour urine free cortisol (24-h UFC), high- and low-dose dexamethasone suppression tests; (7) the electrocardiogram and echocardiography findings; (8) the treatment of hypercortisolemia; and (9) the outcomes after the diagnosis and treatment.

#### Literature Review of CS Complicated With Cardiomyopathies

The literature search of the PubMed online database was conducted up to August 1, 2021; we searched for keywords including [Cushing syndrome], [Hypercortisolism], and [cardiomyopathy]. The inclusion and exclusion criteria were the same as those mentioned above.

Twenty patients from 13 studies had CS complicated with cardiomyopathies, of which 8 patients from 1 study (20) were excluded due a lack of detailed clinical data. Finally, 12 patients met the inclusion and exclusion criteria, and relevant medical information was collected.

#### Cases of Cushing Disease Without Cardiomyopathies

Since most cases of CS complicated with cardiomyopathies were ACTH-dependent CS in our medical center, 242 CD cases without cardiomyopathies who had detailed clinical evaluation of cardiac structure and function were collected from the hospital information system of Peking Union Medical College Hospital for comparison. All the 242 cases were admitted to the inpatient department and had through cardiac evaluation including echocardiography, showing no evidence of cardiomyopathy.

### Statistical Methods

Qualitative data are presented as frequencies and percentages. Quantitative data are presented as the mean ± standard deviation if conforming to a normal distribution or medians (25th, 75th percentile). Independent two-sample *t*-tests, chi-square tests, or Mann-Whitney *U*-tests were used to evaluate the statistical significance. Statistical analysis was performed using GraphPad Prism 8 (GraphPad Software, La Jolla, California, USA). *P* < 0.05 indicated significance.

## Results

A total of 19 patients who had a diagnosis of CS complicated with cardiomyopathies were identified in our center [Case 1 to Case 7 (*n* = 7)] and in the literature [Case 8 to Case 19 (*n* = 12)]. A total of 242 patients diagnosed with CD without cardiomyopathies were identified as controls.

### The Demographic of CS Complicated With Cardiomyopathies

The 19 patients with CS complicated with cardiomyopathies included 10 males (52.63%) and 9 females (47.37%), and there was a males to females ratio of 1.11:1. The mean age at diagnosis of CS with cardiomyopathies was 35.33 ± 19.75 years old. The average BMI was 28.78 ± 5.178 kg/m^2^ (*n* = 11) (Table 1).

**Table 1:** Clinical characteristics of CS complicated with cardiomyopathies.

	**Case 1**	**Case 2**	**Case 3**	**Case 4**	**Case 5**	**Case 6**	**Case 7**	**Case 8**	**Case 9**	**Case 10**	**Case 11**	**Case 12**	**Case 13**	**Case 14**	**Case 15**	**Case 16**	**Case 17**	**Case 18**	**Case 19**
Sex	F	M	F	M	M	M	M	F	F	F	F	F	M	M	F	M	M	F	M
Age (year)	30	59	26	31	25	28	20	48	43	28	50	67	20	43	63	64	26	7 (weeks)	4 (months)
BMI (kg/m^2^)	29.6	31.3	28.58	27.9	38.3	25.9	22.5	NA	35.5	26.5	NA	NA	30.12	NA	NA	NA	NA	NA	20.43
Cause of cushing syndrome	Pituitary adenoma	Pituitary adenoma	Pituitary adenoma	Pituitary adenoma	NA	Lung carcinoid	NA	Adrenal adenoma	Adrenal adenoma	Adrenal adenoma	Pituitary adenoma	Adrenal adenoma	Pituitary adenoma	Pituitary adenoma	Adrenal adenoma	SCLC	Pituitary adenoma	Adrenal adenoma	Adrenal adenoma
Type of cardiomyopathies	DCM	HCM	HCM	DCM	DCM	DCM	DCM	DCM	DCM	DCM	DCM	DCM	DCM	DCM	DCM	DCM	DCM	HCM	HCM (Obstructive)
Symptoms of cardiomyopathies	Dyspnea cough	No	Edema of lower extremities	Dyspnea, edema	Cough, fatigue and edema	Fatigue and edema	Dyspnea, cough and edama	Dyspnea	Dyspnea	Dyspnea and cough	No	No	Dyspnea	Dyspnea	No	Dyspnea	Dyspnea exhaustion	Edema	No
Time between the onset of CS and presence of cardiac symptoms (months)	96	NA	24	27	16	8	9	NA	30	17	NA	NA	NA	NA	NA	NA	NA	NA	NA
**Complications**																			
Hypertension	No	Yes	Yes	Yes	No	Yes	Yes	No	Yes	No	No	Yes	No	Yes	NA	Yes	No	Yes	Yes
Diabetes	No	No	No	No	No	No	Yes	NA	NA	No	Yes	Yes	NA	NA	NA	Yes	No	Yes	NA
Hyperlipidemia	Yes	Yes	Yes	Yes	Yes	Yes	Yes	NA	NA	No	No	Yes	NA	NA	NA	NA	No	NA	NA
Hypokalemia	No	No	Yes	No	No	Yes	Yes	NA	NA	No	NA	Yes	NA	Yes	NA	Yes	No	NA	NA
Systolic blood pressure (mmHg)	140	160	181	160	140	190	160	130	150	140	NA	173	120	NA	NA	189	NA	120	110
Diastolic blood pressure (mmHg)	100	100	121	100	90	120	120	85	80	80	NA	116	90	NA	NA	101	NA	80	65
NYHA	II	I	I	II	III	III	II	NA	NA	III	NA	III	NA	NA	NA	NA	NA	NA	I
Treatments to relieve hypercortisolemia	Pituitary surgery	Repeated Pituitary surgerys	Repeated Pituitary surgerys	Pituitary surgery	No surgery due to severe heart failure	Failure pituitary surgery and radiotherapy, then pulmonary carcinoid resection	Pituitary surgery and bilateral adrenalectomy	Drug plus adrenal adenoma resection	Adrenal resection (left)	Drug plus adrenal resection (right)	Drug plus radiotherapy	Adrenal resection	Pituitary surgery	Drug	Adrenal resection	Drug plus chemotherapy	Pituitary surgery	Drug plus adrenal surgery	Adrenal surgery
Number of treatment methods	1	2	2	1	0	3	2	2	1	2	2	1	1	1	1	2	1	2	1
Outcome of cardiomyopathies in the followup	NA	No significant change	NA	NA	No significant change	Improvement	Death	Improvement	Improvement	Improvement	Improvement	Improvement	Improvement	No significant change	Improvement	Improvement	Improvement	Improvement	Improvement

*F, Female; M, Male; DCM, dilated cardiomyopathies; HCM, hypertrophic cardiomyopathies; SCLC, small-cell lung cancer; NYHA, New York Heart Association classification for congestive heart failure*.

The causes of CS with cardiomyopathies were pituitary adenoma (*n* = 8, 42.11%), adrenal adenoma (*n* = 7, 36.84%), ectopic ACTH syndrome (*n* = 2, 10.53%), and unclear causes (*n* = 2, 10.53%). The types of cardiomyopathies were DCM (*n* = 15, 78.95%) and HCM (n=4, 21.05%). At the same time, 11/18 (61.11%) patients also had hypertension, 5/12 (41.67%), diabetes, 8/11 (72.72%), hyperlipemia, and hypokalemia 6/12 (50.00%) (Table 1).

The cardiac symptoms of these patients were mainly dyspnea (10/19), and 5 patients had no relevant symptoms when the cardiomyopathies were detected by ultrasonography. Based on the cardiac function standard of the New York Heart Association, 3/10 patients were grade I, 3/10 were grade II, and 4/10 were grade III (Table 1).

### The Manifestation of Electrocardiography and Echocardiogram or Cardiac MRI

In the available electrocardiograph (ECG) examination, 7/13 patients presented with sinus tachycardia, 1/13 patients presented with atrial premature beat, 1/13 patients presented with ventricular premature beat, 1/13 patients presented paroxysmal atrial fibrillation, and 3/13 patients had no arrhythmia. A total of 2/8 patients had prolonged QTc intervals (469 and 471 ms). ECG ST-T changes were observed in 6/9 patients.

In patients with DCM, the left ventricular end diastolic dimension (LVDd) ranged from 56 to 79 mm, with an average of 63.77 mm (*n* = 10); the interventricular septum dimension (IVSd) ranged from 7 to 13 mm, with an average of 10.40 mm (*n* = 9), among which 3 patients had thickened IVSd; the left ventricular posterior wall dimension (LVPWd) ranged from 7 to 11 mm with an average of 9.4 mm (*n* = 7); and the LVEF ranged from 10 to 50% with an average of 30.39% (*n* = 14). In the HCM patients, the IVSd ranged from 16 to 22 mm with an average of 18.67 mm (*n* = 3); the LVPWd was 10 and 11 mm; the LVDd was 53 mm and 50 mm; and the LVEF was 70% and 69%, which was within the normal range, respectively, in Case 2 and Case 3 (Table 2).

**Table 2:** Echocardiography or cardiac magnetic resonance imaging of cases with CS complicated with cardiomyopathies.

	**Case 1**	**Case 2**	**Case 3**	**Case 4**	**Case 5**	**Case 6**	**Case 7**	**Case 8**	**Case 9**	**Case 10**	**Case 11**	**Case 12**	**Case 13**	**Case 14**	**Case 15**	**Case 16**	**Case 17**	**Case 18**	**Case 19**
LA (mm)	44	41	28	44	66 × 49	47	57 × 52 × 48	NA	NA	NA	NA	NA	NA	NA	NA	NA	NA	NA	NA
LVDd (mm)	61	53	50	56	79	70	68	NA	NA	65.7	NA	56	NA	64	61	57	NA	NA	NA
IVSd (mm)	9	22	16	12	10	7	11	NA	NA	8.6	NA	13	NA	NA	12	11	NA	NA	18
LVPWd (mm)	9	10	11	10	8	7	11	NA	NA	9.8	NA	NA	NA	NA	NA	11	NA	NA	NA
LVEF (%)	33	73	77.1	50	31	28	26	27	45	34	NA	36	20	28–30	24	31	10	NA	NA
FS (%)	16	NA	NA	27	15	14	9.5	NA	NA	NA	NA	NA	NA	NA	NA	NA	NA	NA	NA

*LA, left atrium dimension; LVDd, left ventricular end diastolic dimension; IVSd, interventricular septum dimension; LVPWd, left ventricular posterior wall dimension; LVEF, left ventricular ejection fraction; FS, (Left ventricular) fractional shortening*.

### The Outcomes After Treatment

Among the patients with CS complicated with cardiomyopathies, 8 patients underwent pituitary surgery, and among them, in Case 2 and Case 3, a repeated pituitary operation was performed due to the failure of the first pituitary operation. In Case 6, pulmonary carcinoid leading to excessive ectopic ACTH secretion was diagnosed by further examination after the failure of the first pituitary operation and pituitary radiotherapy, and a resection was performed. In Case 7, a bilateral adrenalectomy was performed due not achieving remission after the pituitary operation and the continued severe hypercortisolism. Seven patients underwent adrenal surgery, and among them, hypercortisolemia drugs were administered prior to surgery in Case 8, Case10, and Case 18. One patient (Case 14) was given medications for hypercortisolemia; 2 patients were administered radiotherapy (Case 11) or chemotherapy (Case 16) after drug therapy; and 1 patient (Case 5) was not treated and was given the recommendation to continue follow up due to the unclear cause of CS and the presence of cyclic hypercortisolism (Table 3).

**Table 3:** Outcomes of cases with CS complicated with cardiomyopathies.

	**Case 1**	**Case 2**	**Case 3**	**Case 4**	**Case 5**	**Case 6**	**Case 7**	**Case 8**	**Case 9**	**Case 10**	**Case 11**	**Case 12**	**Case 13**	**Case 14**	**Case 15**	**Case 16**	**Case 17**	**Case 18**	**Case 19**
ACTH post treatment (pg/ml)	NA	5.3	42.7	12.1	NA	<5	768	NA	NA	NA	NA	NA	8.3	NA	NA	NA	NA	NA	NA
Cortisol post treatment (μg/dl)	0.96	0.7	25.35	7.7	NA	0.62	13.58	NA	NA	NA	NA	NA	0.238	Normal	NA	NA	NA	NA	NA
Improvement time post treatment	–	–	–	–	–	33	–	12	12	6	216	6	18	–	12	2	3	1	10
Change of LVDd	NA	53–48	NA	NA	79–77	70–58	68–71	Normal	NA	65.7–49	NA	56–57	NA	NA	61–51	Normal	NA	NA	NA
Change of IVSd	NA	22–20	NA	NA	10–7	7–7	11–8	Normal	NA	8.6–8.9	NA	NA	NA	NA	12–8	NA	NA	NA	18–6
Change of LVPWd	NA	10–12	NA	NA	8–8	7–8	11–11	Normal	NA	9.8–8.7	NA	NA	NA	NA	NA	NA	NA	NA	NA
Change of LVEF	NA	73–69	NA	NA	31–30	28–57	26–26	27–69	45–60	34–67	?−58	36–51	20–53	29–31	24–50	31–50	10–30	NA	NA

*The symbol ? indicates that pre-treatment data for hypercortisolemia are not available*.

In 12/16 (75.0%) patients who had a follow-up examination, the cardiac structure and function were significantly improved or even completely reversed after remission of hypercortisolemia. Three patients (Case 2, Case 5, and Case 14) showed no significant improvement. Case 2 was a 59-year-old man diagnosed with CD and HCM, who developed cortisol remission after repeated pituitary surgery. Case 5 was a 25-year-old male with CS due to an unknown cause, which was complicated with DCM, who did not undergo surgery due to severe heart failure. Case 14 was a 43-year-old male diagnosed with CD complicated with DCM who did not undergo surgical treatment due to severe heart failure. He was treated with two drugs (metyrapone and ketoconazole) to normalize his cortisol, but myocardial infarction occurred during the follow-up, which led to a rupture of the mitral papillary muscle and mitral regurgitation. A 20-year-old male patient (Case 7) with DCM with an unclear etiology of CS underwent unsuccessful pituitary surgery. Then, a bilateral adrenalectomy was performed to treat the hypercortisolemia. He suffered from sudden cardiac arrest and died 22 days after the adrenal surgery (Table 3).

Ten of 13(76.9%) patients with DCM improved significantly and even completely recovered in regard to their cardiac structure and function after cortisol remission. Their LVEF increased by 25.78 ± 9.28% on average (15–42%), and the median improvement time was 12 months (5.25–21.75 months). One patient (Case 19) with HCM achieved reduction of 12 mm in the IVSd and had cardiac structure normalization within 10 months, while another patient (Case 18) recovered completely with regard to the symptoms of CD, including improvement in HCM just 1 month after surgery.

### Comparison Between CS Patients With and Without Cardiomyopathies

To investigate whether there were some clinical features related to cardiomyopathies due to CS, a comparison was made between ACTH-dependent CS patients with and without cardiomyopathies in our center. The ACTH-dependent CS complicated with cardiomyopathy included 8 patients with CD (Case 1, Case 2, Case 3, Case 4, Case 11, Case 13, Case 14, Case 17) and 2 patients with ectopic ACTH syndrome (Case 6 and Case 16). Compared with CS patients without cardiomyopathies, the proportion of males and the proportion of hyperlipidemia were significantly increased in the CS with cardiomyopathies group (*p* = 0.005 and *p* = 0.0119, respectively). There were no significant differences between the two groups in age (*p* = 0.4708), BMI (*p* = 0.0864), the proportion of patients with hypertension (*p* = 0.2735), or the proportion of patients with diabetes (*p* = 0.6430). The median time from CS symptoms to symptoms/diagnosis of cardiomyopathies was 25.50 (12.00–78.75) months in 4 patients (Table 4).

**Table 4:** Comparison between patients with CS complicated with cardiomyopathies and CD patients without cardiomyopathies.

	**CS complicated with cardiomyopathies (n=10)**	**CD without cardiomyopathies (*n* = 242)**	***P*-value**
Sex, M (%)	7 (70.00)	42 (17.36)	0.0005^*^
Age, year	30.50 (26.00–52.25)	32.00 (24.75–40.25)	0.4708
BMI, kg/m^2^	29.09 (27.40–30.42)	26.90 (24.55–28.82)	0.0864
Course of disease (months)	25.50 (12.00–78.75)	36.00 (12.00–72.00)	0.7010
Hypertension, *n* (%)	6/10 (60.00)	183/242 (75.62)	0.2735
Diabetes, *n* (%)	2/8 (25.00)	44/241 (18.26)	0.6430
Hyperlipidemia, *n* (%)	5/7 (71.43)	57/240 (23.75)	0.0119^*^
Cortisol (μg/dl)	35.48 (28.26–47.10)	28.19 (22.78–34.96)	0.0896
ACTH (pg/ml)	109.00 (91.78–170.30)	68.60 (47.85–110.00)	0.0199^*^
24-hUFC (μg)	729.40 (234.30–821.20)	456.80 (230.10–782.90)	0.3420
Inhibition rate of high dose dexamethasone suppression test (%)	92.37 (87.03–96.45)	87.00 (71.97–95.5)	0.3999
White blood cells (×10^9^/L)	9.58 (8.64–12.38)	8.42 (6.98–10.20)	0.2100
Platelets (×10^9^/L)	150.00 (110.50–253.50)	254.50 (214.50–295.30)	0.0249^*^
Hemoglobin (g/L)	134.50 (116.50–153.00)	144.00 (134.00–154.00)	0.2448
Hematocrit (%)	46.20 (40.70–48.10)	42.40 (40.00–45.70)	0.2524
Serum sodium (mmol/L)	145.50 (140.50–148.00)	141.00 (140.00–143.00)	0.0316^*^
Serum potassium (mmol/L)	2.70 (2.40–3.60)	3.90 (3.50–4.20)	0.0014^*^
Triglycerides (mmol/L)	2.42 (1.52–3.39)	1.53 (1.09–2.37)	0.1442
Total cholesterol (mmol/L)	8.22 (5.26–8.49)	5.79 (4.86–6.52)	0.1234
Low density lipoprotein cholesterol (mmol/L)	5.98 (3.18–6.28)	3.62 (2.96–4.25)	0.1009
Fasting plasma glucose (mmol/L)	4.56 (4.05–6.40)	4.80 (4.50–5.40)	0.5889
Hypersensitive C-reactive protein (ng/L)	0.47 (0.16–1.30)	0.31 (0.0013–3.30)	0.8344

*M, Male*;

* *P < 0.05 indicated significant difference*.

The median level of morning serum cortisol in CS complicated with cardiomyopathies was 35.48 (28.26–47.10) μg/dl (*n* = 6), and in CS patients without cardiomyopathies, it was 28.19 (22.78–34.96) μg/dl. There was no significant difference (*p* = 0.0896). The median ACTH was 109.0 (91.78–170.30) pg/ml in the CS complicated with cardiomyopathies group (*n* = 7), which was significantly higher than that in CS patients without cardiomyopathies, which was 68.60 (47.85–110.00) pg/ml (*p* = 0.0199; Table 4).

The 24 h UFC level, and high-dose dexamethasone suppression test rate were 729.40 (234.30–821.20) μg (*n* = 7), and 92.37% (87.03–96.45%) (*n* = 3), respectively, in the CS complicated with cardiomyopathies group, while those in CS patients without cardiomyopathies were 456.8 (230.1–782.9) μg, and 87.00 (71.97–95.5) %, respectively. There were no statistically significant differences in the 24-h UFC or high-dose dexamethasone suppression rates between the two groups (*p* = 0.3420 and *p* = 0.3999, respectively) (Table 4).

In addition, there were no significant differences in the white blood cell count (*p* = 0.2100), hemoglobin concentration (*p* = 0.2448) or hematocrit (*p* = 0.2524) between the two groups. The platelet levels in CS patients complicated with cardiomyopathies were significantly lower than those in CD patients without cardiomyopathies [150.00 (110.50–253.50) × 10^9^/L vs. 254.5 (214.5–295.3) × 10^9^/L] (*p* = 0.0249; Table 4).

The serum sodium concentration was higher in the CS complicated with cardiomyopathies group [145.50 (140.50–148.00) mmol/L vs. 141.00 (140.00–143.00) mmol/L] (*p* = 0.0316), while the serum potassium concentration was significantly lower than CD patients without cardiomyopathies [2.70 (2.40–3.60) mmol/L vs. 3.90 (3.50–4.2) mmol/L] (*p* = 0.0014; Table 4).

There were no significant differences in triglycerides (*p* = 0.1442), total cholesterol (*p* = 0.1234), low-density lipoprotein cholesterol (*p* = 0.1009), fasting blood glucose (*p* = 0.5889) or hypersensitive C-reactive protein (*p* = 0.8344) between the two groups (Table 4).

## Discussion

Generally, CS is more common in females and has a males to females ratio of ~1:3–4. However, in the 19 CS cases complicated with cardiomyopathies collected in this paper, the ratio of males to females was 1.11:1, which showed no sex difference. Based on the existing data and research, it is difficult to explain the gender difference in this rare phenomenon at present time. The highest incidence of CS was in patients aged between 20 and 40 years old. In this case series, the mean age of patients was 35.33 ± 19.75 years old, which was consistent with that of patients with common CS.

It is well-known that the most common cause of CS is pituitary corticotropin adenoma, namely, Cushing's disease, followed by ectopic ACTH syndrome and ACTH-independent Cushing's syndrome caused by adrenal lesions. In this study, the most common cause of CS was pituitary corticotropin adenoma, followed by adrenal adenoma, and ectopic ACTH syndrome, which was similar to common CS. However, among the cases in the literature, there were more adrenal lesions, with a prevalence of 58.33% and there was an additional 8 CS patients with cardiomyopathy who were not included in this study due to lack of clinical data. But in our case series, both of the Cushing's disease and adrenal adenoma were included to investigate whether they complicated with the cardiomyopathy. And it was found the Cushing's disease was the major etiology type in our center, which means that CS with cardiomyopathy may be due to the different etiologies of CS. There were still requirements for larger studies to determine whether patients with Cushing's syndrome with adrenal lesions are more likely to develop cardiomyopathies.

The prevalence of DCM and HCM differs greatly in various studies. Data have shown that the prevalence of DCM is 1/2,500 (21, 22), and that the HCM prevalence is 1/500 in general population (23, 24). In addition, the prevalence of DCM in Europe and North America was 36/100,000 (22, 25), which was significantly higher than that in East Asia (for example, 14/100,000 in Japan) (26), and the prevalence of HCM in Eurasia and North America was 200–500/100,000 (27–29). In addition, the prevalence of DCM and HCM in China was 19/100,000 and 80/100,000, respectively (30). These data showed that the prevalence of HCM was higher than that of DCM. There are no epidemiological data on the prevalence of CS complicated with cardiomyopathy since it is a rare type of disease. In this study, CS complicated with DCM was more common than with HCM, with a ratio of 3.75:1, which was in contrast to most epidemiological data for common cardiomyopathies regarding the prevalence of HCM and DCM.

Among the CS complicated with DCM, Case 4, Case 6, and Case 7 had only a year hypertension history and had no other conditions that could lead to an enlarged left ventricle and decreased myocardial systolic function, such as valvular heart disease or congenital or ischemic heart disease. In addition, although the LVDd and LVEF in Case 4 were critical, a further review of the data revealed that the patient's LVPd was 63 mm 2 months prior to this, which was treated with anti-heart failure treatments. The echocardiogram showed moderate mitral regurgitation without abnormal valve structure and no opening/closing abnormalities in Case 1, which indicated relative mitral insufficiency due to an enlarged left ventricle. Case 5 and other patients in the literature did not have concomitant risk factors for ventricular dilation, decreased cardiac function, and/or cardiomyopathy confirmed by echocardiography, cardiac MRI, etc. Therefore, these patients were diagnosed with CS complicated with DCM.

DCM mainly occurs in young people and is more common in males, is asymptomatic in the early stage and gradually progresses to heart failure. In this study the ratio of males to females with CS complicated with DCM was 1.14:1, which was not a significant sex difference. In a cohort study of 3,078 hospitalized heart failure patients in Denmark and Sweden, patients with DCM were 10 years younger (median age: 64 years) and had more severe conditions than patients with heart failure due to other causes (31). In this study, the mean age of CS patients complicated with DCM was 39.07 years old, which was younger than that of patients with common DCM. The prognosis of DCM is quite poor. Prazak et al. (32) reported that 52 patients with idiopathic DCM had 1-year, 5-year and 10-year survival rates of 89, 48, and 30%, respectively. Due to pharmacological and non-pharmacological therapeutic strategies, earlier diagnosis, and individualized long-term follow-up and continuous risk refinement, the prognosis of patients with DCM has improved significantly over the past few decades with survival free from heart transplantation rising to more than 80% at 8-year follow-up (33). However, a complete recovery of the cardiac structure and function is unusual, which only occurs in patients in which the acute injury does not cause significant myocardial damage (34). However, among the patients with CS combined with DCM, 10/13 (76.9%) patients showed a significant improvement or even a complete reversal of the heart structure and function after remission of hypercortisolemia after treatment with CS. The average LVEF of CS patients complicated with cardiomyopathies increased by 25.78% at a median time of 12 months after recovery from hypercortisolism. The prognosis of CS with DCM was much better than that of common DCM. It is suggested that the cause of CS complicated with DCM is acquired, and the patient may have a recovery, which could be related to the treatment of the hypercortisolemia.

**Figure 1:**
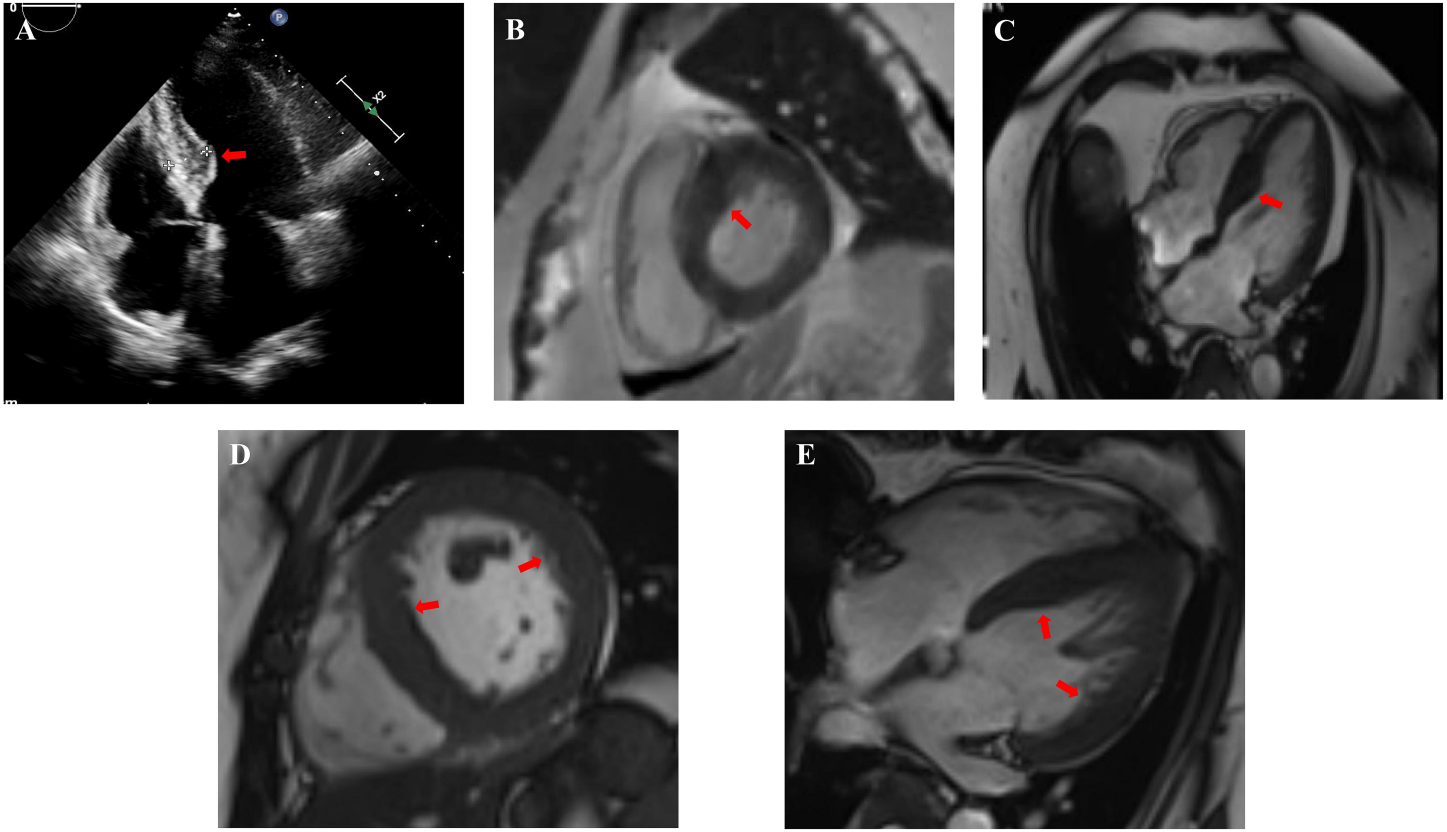
Cardiac images indicated non-uniformly thickened ventricular walls and hypertrophic cardiomyopathy of Case 2 **(A–C)** and Case 3 **(D,E)**. **(A)** Echocardiogram in Case 2 showed significant thickening of the base of the interventricular septum. **(B,C)** Cardiac MRI in Case 2 showed the lateral wall of the left ventricle was about 8–10 mm and the thickening of the basal and intermediate segments of the interventricular septum was uneven with the thickest part about 20–22 mm (red arrow). **(D,E)** Cardiac MRI in Case 3 showed the lateral wall of the left ventricle was about 9–11 mm and the interventricular septum thickened, about 14–16 mm. The red arrows indicate non-uniformly thickened ventricular wall.

In this study, 4 CS patients with HCM were included. HCM is characterized by asymmetrical but sometimes symmetrical ventricular hypertrophy, and the systolic function is mostly but not all maintained, while the diastolic function is decreased to varying degrees. Other conditions causing ventricular hypertrophy should be ruled out before the diagnosis of HCM. Most of HCM are hereditary cardiomyopathies. Genetic studies have shown that HCM is caused by dominant mutations in 11 or more genes encoding the contractile myofilament protein components of the sarcomere or the adjacent Z-disc. Approximately 70% of people have mutations in two genes, the β-myosin heavy chain (MYH7) and myosin binding protein C (MYBPC3) (35–38). HCM also usually occurs in adolescents and young adults, but a growing number of children with HCM are being identified at a young age (<10 years, including in infancy), and adults survive to advanced ages (>80 years) (39, 40). The ages of 2 patients in our center were 59 years (Case 2) and 26 years old (Case 3). Although they had a past history of hypertension, their blood pressure was maintained with the normal range with oral antihypertensive medicine. This means that the HCM was unlikely to be affected by hypertension. Neither of them had diabetes, hyperlipemia, or coronary artery disease. Above all, there was a large difference between the IVSd and LVPWd of Case 2 and Case 3, indicating uneven ventricular wall hypertrophy, and cardiac MRIs suggested the presence of hypertrophic cardiomyopathy (Figure 1). The other 2 patients with HCM (Case 18 and Case 19) in the literature were newborns, and they did not mention other risk factors for HCM for a long enough time.

HCM is one of the leading causes of sudden death in adolescents and during exercise. A study suggested that the annual mortality rate was ~1% for HCM (39, 41). In the present study, Case 18 and Case 19 recovered completely immediately after remission of hypercortisolemia, while Case 2 had no improvements in cardiac structure after 2 years of remission of hypercortisolism. Case 2 was a 59-year-old male who achieved the remission of hypercortisolemia after the repeated pituitary surgery, that was 6 years after the first pituitary surgery. And he also had hypertension for 10 years, which may affect the improvement of cardiomyopathy. He had no family history of HCM and developed HCM after the occurrence of CS. The gene panel test for HCM was also completed with negative result. So it should be considered that his cardiomyopathy was closely related to hypercortisolemia. In addition, a report showed that a male newborn was diagnosed with hypertrophic obstructive cardiomyopathy. His cortisol level was 4.34 μg/dL, and his mother had CD during pregnancy (42).This special case suggested that CS with HCM was related to the hypercortisolemia, which improved after treatment.

Patients with CS may develop a range of metabolic syndrome conditions, including central obesity, hypertension, insulin resistance, impaired glucose tolerance, hyperglycemia and hyperlipidemia. These factors all have a negative impact on the heart and may promote ventricular hypertrophy, fibrosis and fat deposition, leading to cardiac insufficiency.

However, these metabolic factors do not fully explain the changes in the hearts of patients with CS. Avenatti et al. (43) enrolled 25 patients with CS and 25 controls (9 normotensive and 16 hypertensive patients in each group), and the analysis confirmed that the left ventricular mass and relative wall thickness were increased in CS patients compared with matched controls, but this was not directly related to blood pressure. In the cases of CS complicated with cardiomyopathies in this paper, cardiac function recovered in a short time after hypercortisolemia remission, showing a close relationship with the effect of overproduction of cortisol. Hypertension is not invariably present in CS, LV hypertrophy has also been described in CS patients without hypertension (5).

Therefore, apart from the effects of metabolic changes caused by hypercortisolism, the direct effect of cortisol on the myocardium should be considered. Mineralocorticoid receptors are widely expressed in cardiomyocytes, cardiac fibroblasts, vascular smooth muscle cells, endothelial cells and other cardiovascular system cells and lead to oxidative stress, inflammation, myocardial interstitial fibrosis and so on after activation (44, 45). Cortisol-mediated myocardial fibrosis mediated by activating mineralocorticoid receptors may be an important factor leading to cardiomyopathies.

The study of Frustaci et al. (15) further explained the pathogenesis of CS cardiomyopathies. At the time of diagnosis, cell swelling, myocardial fibrinolysis and partial sarcomere disorder were obvious. One year after adrenal surgery, the cell volume, cytoplasmic density and body tissue showed improvements. Meanwhile, atrogin-1 mRNA was 30 times higher in patients at baseline than in normal controls and returned to normal after adrenal resection. Shortly thereafter, Frustaci et al. (20) observed cardiomyopathy features in 8 patients (4 males and 4 females, mean age 61 ± 4.9 years) with CS caused by adrenal adenoma complicated with DCM. The common features of CS cardiomyopathies were cardiac hyperplasia, myofibrinolysis, and myocardial fibrosis. Atrogin-1 levels were 28 times higher in patients with CS cardiomyopathies than in normal controls and 3.5 times higher than in patients with idiopathic DCM. The atrogin-1 levels returned to normal after adrenal resection and were negatively correlated with LVEF. Elevated plasma cortisol levels could activate the FOXO transcription factor, leading to a significant increase in atrogin-1 and ubiquitin levels (46). However, it was reported that the reduction in the myofiber content of cardiomyocytes was a reversible event, and the atrogin-1 level returned to physiological levels 1 year after adrenal resection and after the normalization of plasma cortisol levels. There was also a concurrent reduction in fibrinolytic cell area from 61 to 22%, paralleling the recovery of cardiac structure and function (20).

Compared with CD patients without cardiomyopathies, there were no significant differences in the course of disease, sex, and age, but CS patients complicated with cardiomyopathies had higher serum sodium levels, lower serum potassium levels, and higher ACTH levels. In addition, although no significant difference was observed, patients with CS complicated with cardiomyopathies had a shorter course of disease and higher serum cortisol levels than CD patients without cardiomyopathies. Although no significant difference was observed, this suggests that patients with CS complicated with cardiomyopathies tend to be more severe and have a greater tendency to be affected by hypercortisolemia.

In conclusion, CS complicated with cardiomyopathies is a very rare clinical type, and the most common cardiomyopathy is DCM, followed by HCM. Cortisol plays an important role in the development of cardiomyopathies in CS, and the cardiomyopathies can be greatly improved with remission of hypercortisolemia.

The number of cases in this study is limited, and a larger study is needed to explain the clinical features and prognosis of CS complicated with cardiomyopathies.

## Data Availability Statement

The raw data supporting the conclusions of this article will be made available by the authors, without undue reservation.

## Ethics Statement

The studies involving human participants were reviewed and approved by the Ethics Committee of Peking Union Medical College Hospital. Written informed consent from the participants' legal guardian/next of kin was not required to participate in this study in accordance with the national legislation and the institutional requirements. Written informed consent was obtained from the individual(s) for the publication of any potentially identifiable images or data included in this article.

## Author Contributions

LLu was responsible for the study concept and design. SM did the statistical analysis and wrote the manuscript. LLi, YW, ZL, HZ, LW, LD, XX, YY, MF, and RW collected clinical specimens. All authors critically revised the paper and approved the final version.

## Conflict of Interest

The authors declare that the research was conducted in the absence of any commercial or financial relationships that could be construed as a potential conflict of interest.

## Publisher's Note

All claims expressed in this article are solely those of the authors and do not necessarily represent those of their affiliated organizations, or those of the publisher, the editors and the reviewers. Any product that may be evaluated in this article, or claim that may be made by its manufacturer, is not guaranteed or endorsed by the publisher.
